# Effects of age-related hearing loss and hearing aid experience on sentence processing

**DOI:** 10.1038/s41598-021-85349-5

**Published:** 2021-03-16

**Authors:** Margreet Vogelzang, Christiane M. Thiel, Stephanie Rosemann, Jochem W. Rieger, Esther Ruigendijk

**Affiliations:** 1 Institute of Dutch Studies, University of Oldenburg, Ammerländer Heerstraße 114-116, 26129 Oldenburg, Germany; 2 Cluster of Excellence “Hearing4all”, University of Oldenburg, Ammerländer Heerstraße 114-116, 26129 Oldenburg, Germany; 3 Biological Psychology, Department of Psychology, Department for Medicine and Health Sciences, University of Oldenburg, Ammerländer Heerstraße 114-116, 26129 Oldenburg, Germany; 4 Applied Neurocognitive Psychology, Department of Psychology, University of Oldenburg, Ammerländer Heerstraße 114-116, 26129 Oldenburg, Germany; 5 Present Address: Department of Theoretical and Applied Linguistics, University of Cambridge, Cambridge, UK

**Keywords:** Cognitive ageing, Neuroscience, Auditory system

## Abstract

Age-related hearing loss typically affects the hearing of high frequencies in older adults. Such hearing loss influences the processing of spoken language, including higher-level processing such as that of complex sentences. Hearing aids may alleviate some of the speech processing disadvantages associated with hearing loss. However, little is known about the relation between hearing loss, hearing aid use, and their effects on higher-level language processes. This neuroimaging (fMRI) study examined these factors by measuring the comprehension and neural processing of simple and complex spoken sentences in hard-of-hearing older adults (*n* = 39). Neither hearing loss severity nor hearing aid experience influenced sentence comprehension at the behavioral level. In contrast, hearing loss severity was associated with increased activity in left superior frontal areas and the left anterior insula, but only when processing specific complex sentences (i.e. object-before-subject) compared to simple sentences. Longer hearing aid experience in a sub-set of participants (*n* = 19) was associated with recruitment of several areas outside of the core speech processing network in the right hemisphere, including the cerebellum, the precentral gyrus, and the cingulate cortex, but only when processing complex sentences. Overall, these results indicate that brain activation for language processing is affected by hearing loss as well as subsequent hearing aid use. Crucially, they show that these effects become apparent through investigation of complex but not simple sentences.

## Introduction

Age-related hearing loss typically affects an older adult's ability to hear high frequencies. This type of hearing loss is often left untreated, with large numbers of hard-of-hearing older adults not using a hearing aid in their daily life. For example, only an estimated 14% of hard-of-hearing people over the age of 50 in the US use a hearing aid^1^ and only an estimated 9% of hard-of-hearing people between age 60 and 69 in Canada use a hearing aid^2^. The consequences of this are largely unknown but highly relevant, as many countries face an aging population and untreated hearing loss is associated with cognitive decline^3–5^ and incident dementia^6^.

Hearing loss also affects the processing of spoken language. While this may sound like a logical consequence of degraded auditory input, the effects seem to go beyond the auditory level, also affecting higher-level linguistic processing. For example, adults with mild-to-moderate hearing loss have problems with the comprehension of complex sentences (especially at higher speech rates^7^), respond slower to comprehension questions about complex sentences^8^, and show increased sentence processing times^9^ compared to normal-hearing peers.

The neural processing of speech typically relies on a network that includes the middle and superior temporal gyrus and the inferior frontal gyrus^10^. Speech processing predominantly takes place in the left hemisphere, which is the dominant hemisphere for language processing, although corresponding areas in the right hemisphere are also part of the network^10^. Neurophysiological research has shown that hearing loss is associated with decreased activity in cortical regions associated with this speech processing network^11,12^ as well as in subcortical regions^11^ during auditory sentence processing. In contrast, hearing loss increases recruitment of regions beyond the traditional speech processing network when processing spoken language, specifically of frontal areas^13,14^. Such increased ecruitment may be a compensatory mechanism, without which communicative abilities would suffer^15,16^. However, compensating for perceptual difficulties caused by degraded input in hard-of-hearing individuals is effortful^17^ and may therefore interfere with other taxing processes, such as higher-level language processing (cf.^18,19^).

Age-related hearing loss can be treated quite effectively through the fitting of hearing aids. Recent research has shown that 3 months after being fitted with a hearing aid, hard-of-hearing subjects report improved speech understanding and decreased listening effort in everyday life^20^. Moreover, hearing aids may alleviate some of the speech processing disadvantages associated with hearing loss. Specifically, hearing aid users compared to non-users show shorter processing times under similar hearing conditions^9,21^, which seem to level out when non-users are fitted with hearing aids for 6 months^22^. Similarly, improvements in speech identification have been found following hearing aid fitting^23,24^.

Regarding the neurophysiological effects of hearing aid use, findings are less clear. A recent study^25^ using EEG source reconstruction found increased activity in auditory, frontal, and pre-frontal areas for hard-of-hearing subjects without hearing aids compared to a control group when processing visual symbols. In addition, more severe hearing loss correlated with increased right auditory activation. The same hard-of-hearing subjects showed a reversal of these effects following the use of bilateral hearing aids for 6 months. In addition, subjects' speech perception in noise improved as a result of using hearing aids. In contrast, Dawes et al.^26^ found no neural effects of hearing aid fitting on the processing of simple tones using EEG measures. Hwang et al.^27^ provided subjects with one hearing aid in order to compare effects on the aided versus the unaided ear. Using fMRI measures, they found decreased activity—compared to a baseline measure before hearing aid fitting—in the superior temporal gyrus following the presentation of speech sounds to the unaided ear as well as the aided ear. Finally, Habicht et al.^28^ used fMRI measures and found decreased activity in superior and middle frontal regions outside of the speech processing network in hearing aid users compared to hard-of-hearing unaided listeners when processing speech in noise. In addition, they found increased activity in the lingual gyri and the left precuneus with longer hearing aid use when processing complex sentences. Since decreased activity in frontal regions has been found when hearing aid use was used as a grouping factor^27,28^, but increased activity in other regions has been found to correlate with length of hearing aid use^28^, both the location and the direction of the influence of hearing aid use on neural processing remain unclear. In addition, many of these studies had small sample sizes (e.g., groups of 8^27^ or 13^28^ participants) and in Habicht et al.^28^ the significance thresholds were not corrected for multiple comparisons. As the authors^28^ acknowledge the accompanying higher risk of false positives and the need for follow-up studies, it is clear that more research on hearing aid use in relation to sentence processing is needed. Thus, even though there are some indications of behavioral as well as neural effects of hearing aid use, the relationship between hearing loss, the use of hearing aids, and their effects on higher-level language processes in relation to neural changes is not well understood yet. In addition, the influence of the duration of hearing aid use on linguistic processing has as of yet not been examined in a structured manner.

In this study, we investigate the correlation between hearing loss severity and hearing aid use on the one hand and the comprehension and neural processing of simple and complex sentences on the other. We used an experimental paradigm in which participants were presented with auditory recordings of simple and complex sentences. Specifically, we manipulated word order to create sentences of different complexities in German. Simple sentences were as in (1), where the subject of the sentence precedes the object and the adjunct is in third position. Complex sentences were derived from that by two word order changes. These two changes are assumed to involve different types of syntactic processing^29,30^. The first change involved putting the object before the subject. This leads to increased processing cost^18,31,32^. The second change involved changing the position of the adverb, from the third position (which is assumed to be the default, canonical position) to the first. Finally, these two word order changes were combined to create, presumably, the most complex structure shown in (2). An overview of all sentence structures is shown in Table 1.

**Table:** 

(1)	Der_NOM_ Hund	berührt	am Montag	den_ACC_ Igel
	The_NOM_ dog	touches	on Monday	the_ACC_ hedgehog
	[Subject]	[Verb]	[Adjunct]	[Object]
	*The* *dog* *touches* *the* *hedgehog* *on* *Monday*

**Table:** 

(2)	Am Montag	berührt	den_ACC_ Igel	der_NOM_ Hund
	On Monday	touches	the_ACC_ hedgehog	the_NOM_ dog
	[Adjunct]	[Verb]	[Object]	[Subject]
	*The* *dog* *touches* *the* *hedgehog* *on* *Monday*

**Table 1:** Example sentences in each of the four sentence conditions (S = Subject, V = Verb, A = Adjunct, O = Object).

Adjunct location	Subject-object order	Word order	Example sentence
Adjunct-third	Subject-before-object	SVAO	Der_NOM_ Hund berührt am Montag den_ACC_ Igel
Adjunct-third	Object-before-subject	OVAS	Den_ACC_ Igel berührt am Montag der_NOM_ Hund
Adjunct-first	Subject-before-object	AVSO	Am Montag berührt der_NOM_ Hund den_ACC_ Igel
Adjunct-first	Object-before-subject	AVOS	Am Montag berührt den_ACC_ Igel der_NOM_ Hund *On* *Monday* *the* *dog* *touches* *the* *hedgehog*

Furthermore, by using different sentence structures, we could reduce the predictability of the upcoming sentence. The sentences therefore require continuous linguistic processing for correct interpretation. Also, some effects of hearing loss or hearing aid use might only emerge when a task is sufficiently difficult, such as the processing of complex sentences [e.g.,^18,31,32^]. Thus, effects of these hearing-related measures may be found during the processing of complex sentences but not during the processing of simple sentences (as in^28^). Individual measures of hearing loss and the duration of hearing aid use (hearing aid experience) were obtained from each participant, so that their associations with sentence processing could be investigated. In addition, a non-verbal secondary task (as part of a dual-task paradigm) was included to increase the load on participants and therefore to potentially elicit stronger effects of hearing loss and hearing aid experience. Dual-task situations are common in everyday life. Crucially, the secondary task (a fixation cross change detection task) was domain-unspecific (i.e. non-auditory, non-linguistic) and therefore increased load in a different domain than the increase caused by complex sentences. Specifically, the task was aimed at increasing demands of visual attention and executive control without taxing working memory or interfering with auditory processing. This way, it can be examined whether sources of processing load in different modalities [domain-specific (sentence complexity) vs. domain-general (secondary task)] are affected by hearing loss and hearing aid experience in different ways.

Based on the literature presented above, we formulated two main hypotheses. Firstly, we expected increased hearing loss to be associated with less successful comprehension of complex sentences, especially in dual task situations. At the neural level, we expected increased hearing loss to be associated with decreased activity in cortical regions related to the speech processing network^11^, and increased activity in frontal regions outside of this network^13,14^ (Hypothesis 1). Secondly, we expected increased hearing aid experience to alleviate some of the effects of hearing loss and to therefore show opposite effects. Based on this, we expected increased hearing aid experience to be associated with improved comprehension of complex sentences, increased activity in cortical regions related to the speech processing network, and decreased activity in frontal regions outside of this network (Hypothesis 2). Based on previous research^28^, we expect the strongest effects of hearing aid experience to emerge in the processing of complex sentences (compared to simple sentences). If this is the case, it would reflect effects in higher-level language processing rather than auditory processing.

## Methods

### Participants

**Figure 1:**
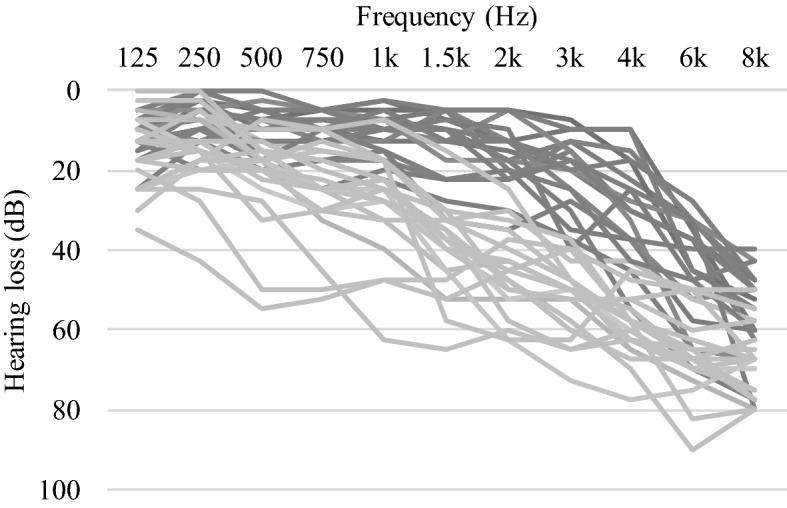
Pure tone audiograms. Average pure tone audiograms for all subjects averaged over both ears (hearing aid users in light grey, non-hearing aid users in dark grey).

39 older adults participated in the study (19 females; mean age 65.5; age range 54–73). All participants showed mild-to-moderate binaural sloping age-related hearing loss with a group-average Pure Tone Average (PTA)-high (the average hearing loss at 2, 4, 6, and 8 kHz) of 48.5 dB HL (see Fig. 1 for audiograms). An overview of the participants' demographic, audiological, and cognitive characteristics is given in Table 2. The group of participants included both hearing aid users who used their hearing aid for a minimum of 4 h daily (n = 19; mean age 65.5 ± 5.1) and non-users who had never used a hearing aid (n = 20; mean age 65.6 ± 4.1). The hearing aid users had been using a hearing aid for an average of 6.1 years (hearing aid experience). Our original idea was to compare groups of hearing aid users and non-users directly, but in Germany it is difficult to find elderly participants with more severe (i.e. moderate) hearing loss who do not already use hearing aids. As a consequence, we were not able to get a sufficient number of participants with similar hearing loss, hence the hearing aid users had more severe hearing loss on average than the participants without hearing aids (PTA-high respectively 60.8 ± 6.1 and 36.8 ± 7.8), rendering a group comparison inappropriate. We therefore decided to investigate the influence of hearing aid use within the group of hearing aid users as a function of the length of use. In addition, the influence of hearing loss severity will be investigated in the combined sample. All participants were right-handed, had normal or corrected-to-normal vision, were native speakers of German, had no contraindications for MRI, and reported no language or neurological disorders. Participants showed normal cognitive functioning at a group level as determined by the Montreal Cognitive Assessment task (mean score of 27.2 out of 30^33^). Informed consent was obtained from all participants prior to the start of the study and all participants received a monetary compensation afterwards. The study was approved by the ethics committee of the University of Oldenburg (“Kommission für Forschungsfolgenabschätzung und Ethik”, approval number Drs. 28/2017) and carried out in accordance with the Declaration of Helsinki.

**Table 2:** Mean values and standard deviations of the demographic, audiological, and cognitive information for the participants (n = 39).

	Mean values (s.d.)	Range (min–max)
Age (years)	65.5 (4.7)	54 to 73
Education (years)	16.4 (4.3)	10 to 28
PTA-high (dB)	48.5 (14.1)	26.3 to 71.9
Listening effort (score)	6.7 (1.9)	5 to 12
OLSA (dB)	76.6 (8.4)	58.6 to 100
Hearing aid experience (years; n = 19)	6.1 (4.5)	1 to 19
MoCA (score)	27.2 (2.2)	22 to 13
Digit Span, backwards (score)	7.1 (2.0)	4 to 12
CTMT (sec)	23.3 (15.8)	− 3.4 to 65.8
Vocabulary (score)	32.8 (3.4)	24 to 39

Listening effort (subjective) was obtained through a questionnaire asking participants how difficult they found it to understand speech during several everyday situations and activities, such as watching TV.

*OLSA* Oldenburg (Matrix) Sentence Test^37–39^.

*MoCA* Montreal Cognitive Assessment task with a maximal score of 30^33^.

*CTMT*  Comprehensive Trail-Making Test, difference between subtests 1 and 5^36^.

*Vocabulary* *Wortschatztest*, a German vocabulary test with a maximal score of 40^40^.

### Materials and design

Sentences were taken from a previous study on the processing of different word orders^34^, based on the German OLACS corpus^35^. All sentences contained a Subject (S), a transitive Verb (V), a temporal Adjunct (A), and an Object (O). The position of the adjunct (first or third) and the order of the subject compared to the object (subject-before-object or object-before-subject) were manipulated. This resulted in four sentence conditions (see Table 1), varying in word order and consequently in sentence complexity. SVAO is the canonical, and hence simple word order in German. OVAS, AVSO, and AVOS are non-canonical, more complex word orders in German, with AVOS arguably being the most complex (see^34^). Finally, a condition without sentences (i.e. a silent condition) was included to serve as a baseline of neural activity in the analyses. A fixation cross was displayed on the screen during sentence (or baseline) presentation.

After the sentences were presented to the participants auditorily over headphones, two pictures were presented for a picture selection task (pictures taken from^34^ based on^9,32^). Both pictures contained both characters, but differed in which character performed the action mentioned in the sentence. More specifically, one picture displayed the correct character performing the mentioned action (i.e. in the examples from Table 1: the dog) on the second character (i.e. the hedgehog). The other picture displayed the exact reversal, namely the second character performing the mentioned action (i.e. in the examples from Table 1: the hedgehog) on the other character (i.e. in this case, the dog). Participants could select the picture that matched the meaning of the sentence best with a button press; left button (right index finger) for the left picture and right button (right middle finger) for the right picture. In the silent baseline condition, participants could select a picture randomly. The experiment included a dual task condition in half of the trials in order to examine the effect of non-modality specific processing load. In this condition, a secondary task had to be performed alongside the primary sentence processing task. In this secondary task, the horizontal or the vertical bar of the fixation cross changed in size exactly once during the sentence presentation. Participants were instructed to press a button when they noticed this change (for more details on this paradigm see^34^).

Each trial consisted of the sentence presentation (3.5 s), the picture selection task (3.5 s), and a jitter of 0.3–0.7 s before and after the sentence presentation. The experiment used a within-subjects design with 240 trials (24 trials per condition with 10 conditions, namely 4 sentence conditions and one baseline condition in both single and dual task condition). These were distributed over two sessions (120 trials each). Each session was subsequently divided into six blocks (20 trials each), which alternatingly presented the single and the dual task condition. Instructions on the screen before the start of each block informed participants about which condition would come next. Two pseudo-randomized test-lists were created to counter any order effects.

Visual stimuli were presented by a projector (DATAPixx2, VPixx Technologies Inc.) on a screen, which was positioned behind the MRI (distance of 50 cm from eye to screen). Stimulus presentation was controlled by Presentation software (version 18.3, NeuroBehavioral Systems, Inc., Berkeley, CA, www.neurobs.com).

### Procedure

The study comprised the linguistic experiment described above as well as various cognitive measurements. Informed consent, questionnaires (asking for age, hearing aid use, listening effort), audiogram measurements, the Comprehensive Trail-Making Test (CTMT;^36^) as a measure of cognitive flexibility, the Digit Span task as a measure of working memory capacity, and a practice for the main experiment were performed outside the MRI scanner. Inside the MRI scanner, noise cancellation was applied via MR compatible headphones (Opto Active, Optoacoustics Ltd, Israel). Participants were tested without hearing aids. The sound intensity of the stimuli from the main linguistic experiment was adjusted to 80% intelligibility for each participant individually using the Oldenburg (Matrix) Sentence Test^37–39^ to correct for participants' hearing and for background noise inside the scanner. A second practice round for the main experiment, the main experiment (two sessions; approx. 18 min each), a structural scan (T1), and resting state and DTI measurements (not reported here) were performed inside the scanner. Between the two sessions of the main experiment, participants had a 10- to 15-min break outside the scanner, in which they completed a German vocabulary test^40^ as a measure of verbal intelligence. The complete study took around three hours.

### Behavioral data analysis

The behavioral data analyses were performed in the statistical computing software R (version 3.6.2, www.r-project.org^41^). The rates of correct responses (dependent variable) were examined for each sentence condition in the single and the dual task. Binomial generalized linear mixed-effect-based models were used to investigate the influence of sentence condition, task condition, PTA-high, and hearing aid experience (hearing aid use in years, only for the 19 participants who were hearing aid users) on the rate of correct responses. The warranted inclusion of fixed factors, as well as that of several covariates (age, digit span, CTMT, vocabulary) was examined by means of model comparisons. A maximal converging random effects structure was used. The best model included *sentence* *condition* (with SVAO as the baseline), *session* (i.e. before vs. after the break), and *vocabulary* as fixed factors. *Task* *condition*, *PTA-high*, *hearing* *aid* *experience*, *age,* *digit* *span,* and *CTMT* did not improve the model. Post-hoc pairwise comparisons (Bonferroni corrected) were applied to examine the differences between each of the sentence conditions. The relation between age on the one hand and PTA-high and hearing aid experience on the other was examined to check whether higher age correlated with more hearing loss and/or longer hearing aid experience, but this was not the case.

### MRI data acquisition

MRI data acquisition was performed with a 3 T Siemens Magnetom Prisma MRI scanner using a 20-channel head coil. T_2_*-weighted gradient echo planar imaging (EPI) with BOLD contrast was used (TR = 1800 ms, TE = 30 ms, flip angle = 75 degrees, df = 20, slice thickness = 3 mm, FOV = 192 cm, 33 slices). 600 whole-brain volumes were acquired in the first session; 617 whole-brain volumes were acquired in the second session (the first 17 scans constituted 4 warm-up trials for participants and were removed to eliminate magnetic saturation effects). Anatomical images were recorded with a 3-D T1-weighted MP-RAGE sequence (TR = 2000s, TE = 2.07 ms, flip angle = 9°, slice thickness = 0.75 mm, 224 slices). The mean signal-to-noise ratio of the two experimental sessions was 2.9 (s.d. = 0.3; calculated using the MRIQC-tool^42^).

### fMRI data analysis

(Pre)processing and analysis was performed in SPM12 (Statistical Parametric Mapping, Wellcome Department of Imaging Neuroscience, University College London, http://www.fil.ion.ucl.ac.uk/spm). Preprocessing steps included motion correction and realignment estimation, coregistration, segmentation, and normalization to the Montreal Neurological Institute (MNI) space using normalization parameters obtained from the segmentation of the anatomical T1-weighted image. In addition, the functional data were smoothed with a Gaussian filter (8 mm FWHM kernel; default smoothing kernel in SPM).

General linear models were used for first-level analyses per participant, applying a high-pass filter of 128 s, accounting for serial correlations with an AR(1) model, and using the six head movement parameters obtained in the realignment estimation as regressors. The four sentence types were modelled as boxcar functions (from sentence onset until the onset of the pictures; 3.5 s + jitter) and convolved with a canonical hemodynamic response function. Since no behavioral effects of task condition (i.e. single vs. dual task) were found, we first examined the differences between sentence processing (i.e. the contrast all sentence conditions > baseline condition without sound) in the two different task conditions in first-level and subsequently second-level comparisons. As the second-level comparison showed no differences between the processing of sentences in the single task condition compared to the dual task condition, we collapsed over the two task conditions in all subsequent analyses. This decreases the number of comparisons and increases their power. Next, planned first-level analyses were used to individually calculate estimates for the contrasts (1) SVAO > baseline (the effects of canonical sentence processing) (2) OVAS > SVAO (the effects of object-before-subject word order), (3) AVSO > SVAO (the effects of adjunct-first word order), and (4) AVOS > SVAO (the effects of adjunct-first, object-before-subject word order). After that, we calculated estimates for the contrast of all sentences together > baseline in order to examine whether sentence processing *in* *general* shows effects of hearing loss or hearing aid experience. Furthermore, we calculated estimates for each of the complex sentences compared to the baseline (i.e. OVAS > baseline, AVSO > baseline, and AVOS > baseline), to check for consistency of results across the different sentence types.

To investigate the relation between the level of hearing loss and sentence processing, we ran F-contrasts at a group level using the first-level estimates, with PTA-high, age, cognitive measures (digit span, CTMT, vocabulary), and a binary dummy variable for hearing aid use entered as covariates. In addition, to investigate the relation between the hearing aid use and sentence processing, we ran separate F-contrasts for the hearing aid users only, with years of hearing aid experience, PTA-high, age, and cognitive measures (digit span, CTMT, vocabulary) entered as covariates. Effects are reported as significant when they exceed a cluster-level (Family-Wise Error) corrected threshold of *p* < 0.05 (with a *p* < 0.001 cluster-forming threshold). When the F-contrasts indicated significant effects of the covariate of interest (hearing loss or hearing aid experience), we followed up with simple t-contrasts to determine the direction of the covariate effect. In addition, a simple t-contrast was performed to examine the neural activity in simple sentence processing compared to the silent baseline (SVAO > baseline) as an overall check of auditory- and language-processing-related activity during canonical sentence processing. All peaks are reported in MNI-space. Localization of the corresponding brain regions was achieved with SPM12 and the Yale BioImage Suite Package (http://sprout022.sprout.yale.edu/mni2tal/mni2tal.html).

Finally, to make sure that the observed effects of hearing aid experience were not driven by extreme cases, we performed additional correlation analyses with Spearman's ρ. This correlation coefficient has low sensitivity to outliers as it limits data points to the value of their rank. These correlations were done with the parameter estimates for each participant extracted from SPM12 and performed within the statistical computing software R.

## Results

### Behavioral results

**Figure 2:**
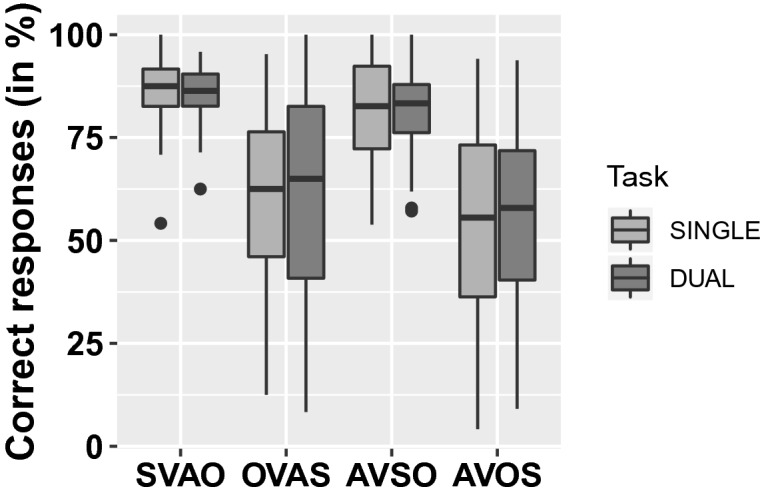
Performance on the picture selection task. Boxplot showing the median and first and third quartile of the percentage of correct responses on the picture selection task as a function of sentence type and load. Statistically significant differences in correct responses were found between all sentence conditions, with performance being highest on canonical SVAO sentences and lowest on AVOS sentences. No significant effect of task condition (single or dual) was found. No significant correlations between performance and PTA-high (hearing loss) or hearing aid experience were found. This figure was created in R (version 3.6.2, www.r-project.org^41^).

The results from the picture selection task, i.e. the sentence comprehension results, are presented in Fig. 2 and Table 3. The results showed effects of sentence condition, with participant performing better on the simple SVAO sentences than on all three types of complex sentences (OVAS *β* = − 1.48; *z* = − 16.39; *p* < 0.001, AVSO *β* = − 0.25; *z* = − 2.54; *p* < 0.05, AVOS *β* = − 1.72; *z* = − 18.99; *p* < 0.001). This shows that the canonical SVAO sentences are easiest to interpret. Based on visual inspection of Fig. 2, the opposite condition, AVOS, with adjunct-first and object-before-subject word order, seems most difficult to interpret. This was confirmed by pairwise comparisons, which showed that the rate of correct responses was different between all complex sentence conditions (all *p*'s < 0.05), with AVOS sentences having the lowest rate of correct responses. Task condition (single or dual), PTA-high, hearing aid experience, age, digit span, and CTMT did not improve the model and therefore we found no evidence of these factors influencing the rate of correct responses. Participants' score on the vocabulary task showed a positive relation with their rate of correct responses (*ß* = 0.09; *z* = 10.82; *p* < 0.001).

**Table 3:** Results for the fixed effects from the binomial generalized linear mixed-effect-based model of the performance on the picture selection task; results from the post-hoc pairwise comparisons between the remaining conditions are presented at the bottom of the table.

Predictors	Estimate	Std. Error	*z* value	*p* value
(Intercept)	− 1.284	0.295	− 4.357	< 0.001
OVAS	− 1.484	0.091	− 16.389	< 0.001
AVSO	− 0.252	0.100	− 2.539	0.011
AVOS	− 1.716	0.090	− 18.991	< 0.001
Session 2	0.332	0.059	5.597	< 0.001
Vocabulary	0.092	0.009	10.815	< 0.001
R^2^** = **21.7%.				
**Additional pairwise comparisons**				
OVAS—AVSO	− 1.231	0.087	− 14.136	< 0.001
OVAS—AVOS	0.233	0.075	3.085	0.011
AVSO—AVOS	1.464	0.087	16.819	< 0.001

SVAO and session 1 were coded as the reference levels.

### Imaging results

The results of the overall check of auditory- and language-processing-related activity during canonical sentence processing in the experiment (main effect of the contrast SVAO > baseline) showed an increase in neural activity related to sentence processing, with a large activation cluster with its peak in the left superior temporal gyrus. These results show that evidence of sentence processing was robustly detected in the data; more detailed results of this comparison are presented in Table S1 and Fig. S1 in the Supplementary Information online.

### Hearing loss

**Figure 3:**
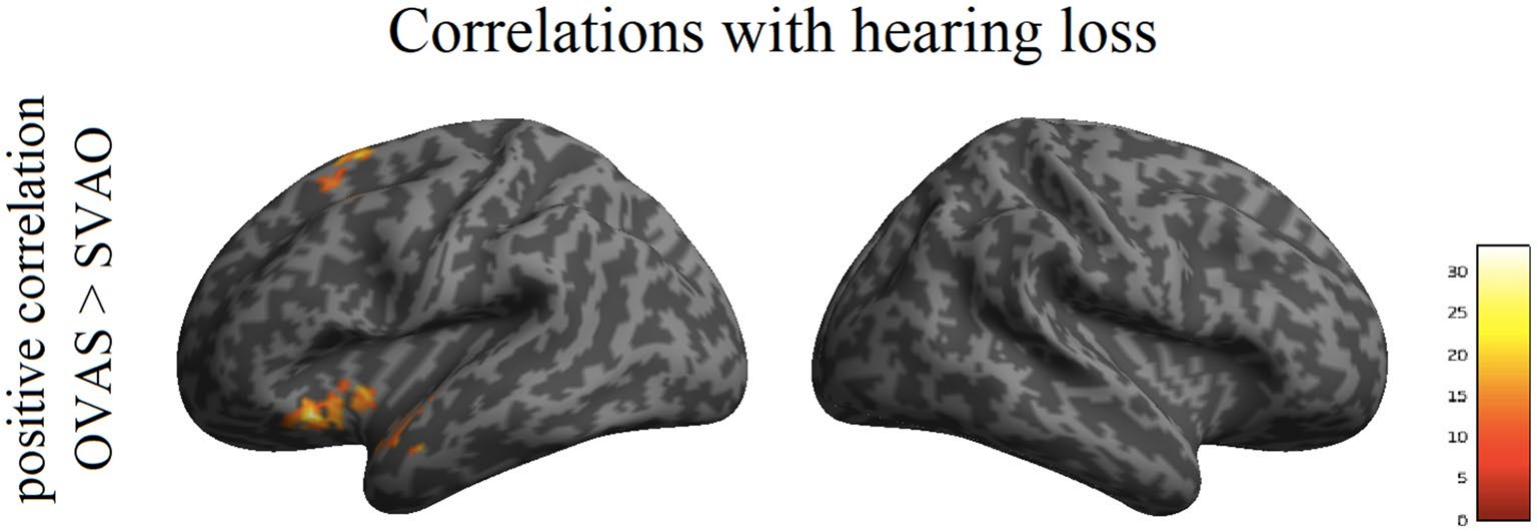
Neural activity associated with hearing loss. Increased neural activity for the processing of object-before-subject word order (OVAS > SVAO) with increasing hearing loss (PTA-high; Family-Wise Error corrected on the cluster level, threshold of p < 0.05). Inflated brain images were created with SPM12 (http://www.fil.ion.ucl.ac.uk/spm); the colors reflect the F values from the F-contrasts.

The potential relationship between level of hearing loss (as defined by PTA-high) and sentence processing was investigated in the contrast between the canonical SVAO sentence and the baseline condition without sound (SVAO > baseline), as well as in the contrasts between the three complex sentence conditions and the canonical sentence condition (i.e. OVAS > SVAO, AVSO > SVAO, and AVOS > SVAO). This enables examining the impact of hearing loss severity on simple sentence processing, as well as its impact on complex compared to simple sentence processing. Correlations between the level of hearing loss and the processing of object-before-subject word order (OVAS > SVAO) were found in the left anterior insula (BA13) and the left superior frontal gyrus (BA6; see Table 4, Fig. 3). Follow-up t-contrasts showed that these correlations were positive, thus showing increased activity for processing the more complex sentences in these regions with more severe hearing loss.

**Table 4:** Maxima of brain regions (MNI coordinates) for the increased neural activity for the processing of object-before-subject word order (OVAS > SVAO) with increasing hearing loss (PTA-high; L = Left; Family-Wise Error corrected on the cluster level, threshold of p < 0.05).

Peak coord. (x,y,z)	Z value	Cluster size	Brain region
(− 32, 18, − 10)	**4.58**	**591**	**L anterior insula**
(− 32, 2, − 6)	3.83		
(− 44, 8, − 8)	3.82		L anterior insula
(− 16, 12, 66)	**4.32**	**256**	**L superior frontal gyrus**
(− 22, 20, 54)	3.52		L superior frontal gyrus
(− 30, 14, 58)	3.51		L middle frontal gyrus

All other comparisons rendered no significant effects of hearing loss. This included the comparisons of all sentences together compared to the baseline and of each of the complex sentences compared to the baseline (i.e. OVAS > baseline, AVSO > baseline, and AVOS > baseline). Interestingly, that means that no effects of hearing loss were found in general sentence processing, simple sentence processing, or in complex sentence processing compared to silence. Rather, the effects only emerge when examining the difference between complex sentence processing (of object-before-subject sentences) compared to simple sentence processing. Importantly, the found effects occur when hearing aid use, age, and cognitive measures are corrected for.

As an additional, exploratory, analysis, we examined whether similar patterns of association between hearing loss and complex sentence processing can be seen in other sentence types. We therefore extracted the parameter estimates (Beta values) for each participant at the two peaks from Table 4 for the contrasts of SVAO > baseline, AVSO > SVAO, and AVOS > SVAO. The significant correlations in the contrast of OVAS > SVAO were also included. The relation between the Beta values in these contrasts and hearing loss are presented in the Supplementary Information online. Supplementary Fig. S2 shows that the positive relation between neural activity and hearing loss as found in the OVAS > SVAO contrast does not appear to be present in the other contrasts.

### Hearing aid experience

**Figure 4:**
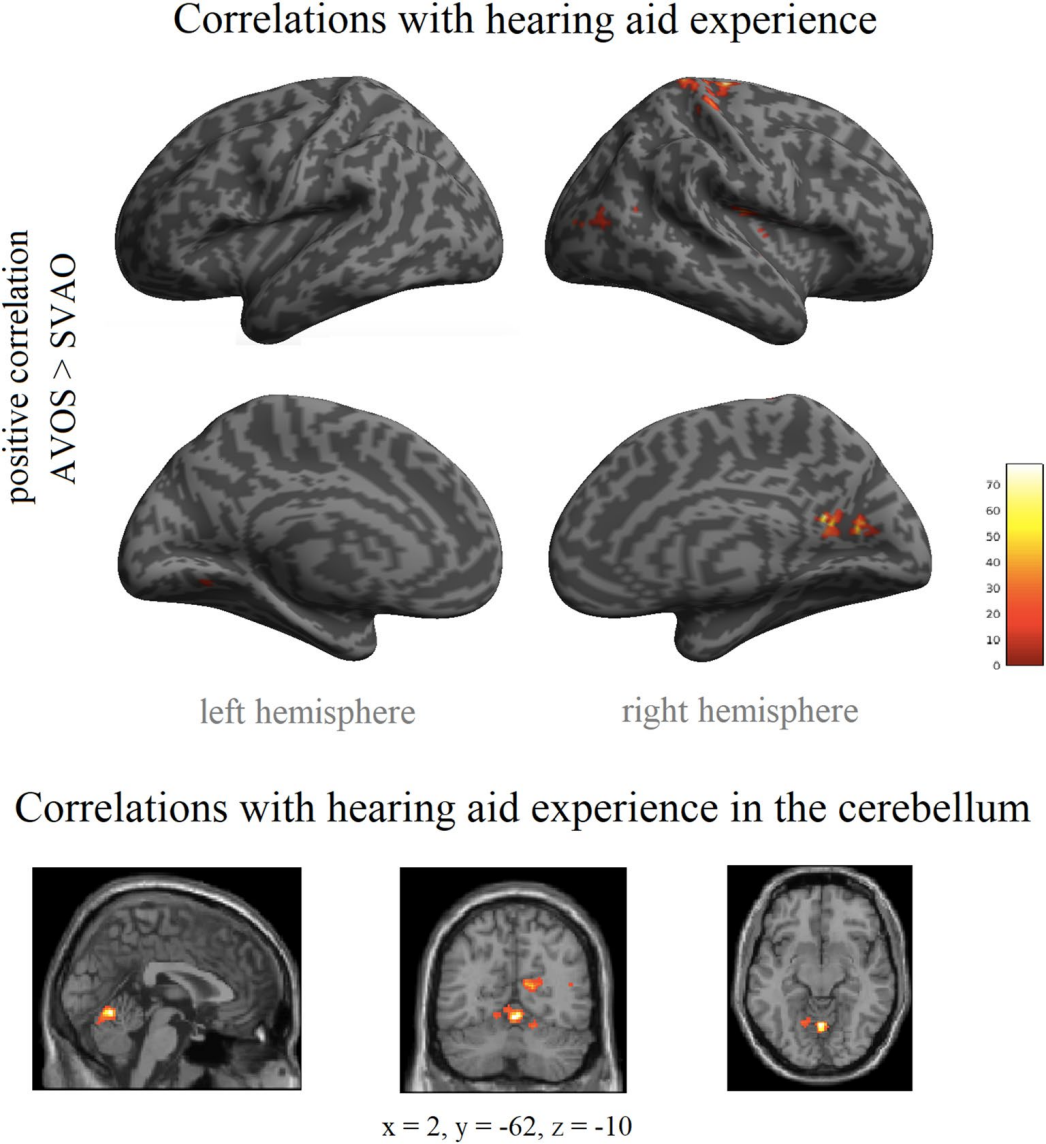
Neural activity associated with hearing aid experience. Increased neural activity for complex sentence processing (AVOS > SVAO) with longer hearing aid experience (i.e., years of hearing aid use) (Family-Wise Error corrected on the cluster level, threshold of p < 0.05). Inflated brain images and slice images were created with SPM12 (http://www.fil.ion.ucl.ac.uk/spm); the colors reflect the F values from the F-contrasts.

The relationship between years of hearing aid experience and sentence processing was investigated, for the 19 participants who were daily hearing aid users, in the same sentence contrasts as above. Correlations between years of hearing aid use and complex sentence processing were found for the adjunct-first, object-before-subject word order (AVOS > SVAO) in several areas outside of the core speech processing areas including the cerebellum, right precentral gyrus, and right central operculum (see Table 5, Fig. 4). Follow-up t-contrasts showed that these correlations were positive, thus showing increased activity with longer hearing aid use.

**Table 5:** Maxima of brain regions (MNI coordinates) for the increased neural activity for complex sentence processing (AVOS > SVAO) with longer hearing aid experience (i.e., years of hearing aid use); R = Right; Family-Wise Error corrected on the cluster level, threshold of p < 0.05).

Peak coord. (x, y, z)	Z value	Cluster size	Brain region
(2, − 62, − 10)	**4.69**	**362**	**Cerebellar vermal lobules I-V**
(14, − 66, − 18)	4.02		R cerebellum exterior
(24, − 68, − 20)	3.70		R cerebellum exterior
(16, − 26, 68)	**4.50**	**469**	**R precentral gyrus**
(18, − 40, 72)	4.05		R postcentral gyrus
(32, − 32, 64)	3.89		R postcentral gyrus
(42, − 10, 14)	**4.50**	**172**	**R central operculum**
(36, − 20, 20)	4.07		R parietal operculum
(52, − 4, 4)	3.88		R central operculum
(8, − 54, 16)	**4.43**	**271**	**R posterior cingulate cortex**
(12, − 64, 14)	4.14		R calcarine cortex
(20, − 66, 14)	3.82		
(38, − 82, 12)	**3.60**	**114**	**R middle occipital gyrus**
(40, − 74, 14)	3.55		
(44, − 58, 14)	3.46		R angular gryus

Scatter plots of the correlation between hearing aid experience and the parameter estimates (Beta values) for each participant at the five peaks from Table 5 are presented in Fig. S3 (top row) in the Supplementary Information. As can be seen in the scatter plots, there are two participants who have considerably longer hearing aid experience (13 and 19 years) than the other participants and could potentially be outliers. To make sure that the observed correlations are not driven by extreme cases, we additionally performed correlation analyses with Spearman's ρ. The results show that the correlations are still significant in the Cerebellar vermal lobules I–V, the right precentral gyrus, right central operculum, and the right posterior cingulate cortex (i.e. the first four peak regions from Table 5) when accounting for outliers. The correlation coefficients at these peaks were respectively 0.56, 0.50, 0.91, and 0.82 (with respective significance values (*p* values) of 0.01, 0.03, < 0.001, and < 0.001). At the last peak region from Table 5, the right middle occipital gyrus, the correlation (coefficient 0.41) did not reach significance when correcting for outliers (*p* = 0.08).

All other relations between different sentence contrasts and hearing aid experience rendered no significant effects. This includes the contrast of the adjunct-first, object-before-subject word order (AVOS) compared to the baseline. As the AVOS > baseline contrast rendered no significant effects of hearing aid experience but the AVOS > SVAO contrast did, this suggests that effects of hearing aid experience can be found only when examining the specific processing differences between simple and complex sentences; that is, general sentence processing does not seem to change with hearing aid experience.

As an exploratory analysis, we additionally examined whether similar, although less salient, patterns of association between hearing aid experience and sentence processing can be seen in other comparisons. To this end, we extracted the parameter estimates (Beta values) for each participant in each contrast at the five peaks presented in Table 5 and present these graphically in relation to their years of hearing aid experience in the Supplementary Information online. As can be seen in Supplementary Fig. S3, there are clear positive relations between neural activity and years of hearing aid experience in the AVOS > SVAO contrast, which is the contrast that showed significant results in Table 5 and Fig. 4, in all five regions. In addition, the other two contrasts reflecting complex sentence processing, OVAS > SVAO and AVSO > SVAO, also show positive trends in all five regions. Thus, similar neural activation patterns emerge for the other complex sentences, although these did not reach significance in the whole-brain analyses. In contrast, canonical sentence processing (i.e. the SVAO > baseline contrast) shows no evidence of a positive relation between neural activity and years of hearing aid experience.

Taken together, these results show effects of hearing loss severity and of hearing aid experience on the neural processing of auditory complex sentences, with hearing loss severity modulating activity mostly in superior frontal areas and the insula and hearing aid experience modulating activity in various regions outside of the core speech processing areas.

## Discussion

We investigated the effects of hearing loss and hearing aid use on higher-level language processes. We did not find any behavioral effects of hearing loss severity or hearing aid experience on the comprehension of simple or complex sentences. In addition, the dual-task paradigm did not influence behavioral or neural sentence processing. At a neural level, however, both hearing loss severity and hearing aid experience correlated with functional activity during sentence processing. More specifically, hearing loss severity was associated with increased activity in left superior frontal areas and the left insula, but only when processing complex object-before-subject sentences. Longer hearing aid experience in turn was associated with recruitment of several areas outside of the core speech processing network, including the cerebellum, right precentral gyrus, and right cingulate cortex, when processing the most complex sentences used in the experiment, adjunct-first object-before-subject sentences.

As stated above, the dual-task paradigm did not influence behavioral or neural sentence processing. Importantly, the secondary task was a perceptual task of fixation cross change detection, which was specifically designed to interact at a domain-unspecific (i.e. non-auditory, non-linguistic) level. Our findings for the dual-task paradigm are not directly in line with previous studies, which report frontal recruitment as reflecting the involvement of domain-general executive functions to compensate for hearing loss^14,15,43^. Notably, previous studies typically used stimuli that interfere or interact directly with the primary task (e.g., auditory noise—as in degraded speech—or a dual speaker). Thus, the frontal activations frequently observed with domain-specific stimuli may reflect domain-specific rather than general cognitive control. This should be taken into account in future research.

### Effects of age-related hearing loss

We expected increased hearing loss to be associated with decreased activity in cortical regions related to the speech processing network, and increased activity in frontal regions outside of this network (Hypothesis 1). In line with the latter expectation, the neuroimaging results showed that individuals with higher levels of age-related hearing loss exhibited increased activity in the left superior frontal gyrus when processing complex object-before-subject sentences. These results show evidence of functional changes in frontal regions related to hearing loss, in line with previous research^13,14^. These findings support the idea that that frontal region recruitment is a cognitive compensatory process in hearing loss^44,45^ and that neural-functional changes in elderly hard of hearing go beyond the auditory cortex^13,46^.

In addition, effects of hearing loss were found in the left anterior insula. Although not part of the core language processing network^10^, the left anterior insula has previously been associated with speech processing^47,48^. It has been suggested that the insula may function as a hub connecting and mediating higher-order cognitive functions in speech and language processing^47^, and as such could play a compensatory role similar to that of the frontal regions. Furthermore, increased activity in the insula has been shown to reflect effortful listening^14,49^. Interestingly, as similar correlations between hearing loss and activity in the insula were absent in the other sentence contrasts in our study, these effects might only become apparent through investigation of specific—grammatically more complex—sentence structures.

Contrary to our expectations, we found no decrease in activity in cortical regions of the speech processing network in relation to hearing loss. Effects in temporal regions are frequently found to be associated with hearing loss, with decreased activity in these areas associated with poorer hearing^11,13^. A possible explanation for the missing decrease in activity may be that in the previously mentioned studies, sound intensity levels of the stimuli were constant across participants. In contrast, we individually determined sound intensity levels for each participant, which led to increased sound intensity levels with increasing hearing loss. Although sound levels are known to drive auditory activation (see Schreiner and Malone^50^ for an overview), a recent study shows that it is actually individuals' perceived loudness that drives this^51^. Specifically, a study in young normal-hearing participants demonstrated that the BOLD signal in several auditory cortex regions seems to be a linear reflection of perceived loudness but not sound intensity level^51^. The absence of effects in cortical regions of the speech processing network in our study therefore potentially suggests that the effects found in earlier studies could be explained by perceptual difficulties rather than linguistic processing. However, this is mere speculation and the question remains open for further investigation. Complicating this issue, hearing loss has been associated with reduced gray matter volume in the primary auditory cortex^11,52^, among other brain volume changes^53,54^ (although some studies found no effects, see^55^).

It should also be noted that in our study, hearing aid users and non-users were combined when investigating the effects of hearing loss, although the use of a hearing aid in daily life was corrected for in the analyses. While this is not uncommon in other research^9,56^, it presents a confound that might explain the absence of results in regions related to the speech network. In addition, larger sample sizes should be investigated in future work to increase statistical power. Overall, our findings reconfirm the compensatory role of the frontal lobe in speech processing for individuals with hearing loss. However, the precise nature of functional changes in the engagement of other regions, specifically the insula and temporal regions, remains to be investigated further, especially in relation to the processing of higher-level language.

Finally, our predictions included the expectation that hearing loss would lead to less successful comprehension of complex sentences based on previous research^7,56,57^. Yet, in our experiment, although sentence comprehension as such was relatively low for more complex sentence conditions, this did not correlate with severity of hearing loss. Potential explanations for this are similar to those discussed in relation to the neural findings: individual adjustments of sound intensity, limited sample size, or the true absence of an effect. Overall, the finding of modulated neural activity in relation to hearing loss severity in combination with the absence of comprehension difficulties suggests that compensatory mechanisms are being applied successfully, limiting the consequences of hearing loss for communication^15,16^.

### Effects of hearing aid experience

Regarding the effects of hearing aid experience, we expected increased activity in cortical regions related to the speech processing network, and decreased activity in frontal regions outside of this network with longer hearing aid experience (Hypothesis 2). The neuroimaging results showed that individuals with longer hearing aid experience exhibited increased activity in several cortical regions outside of the temporal cortex for complex sentence processing. Thus, effects outside of the speech processing network were observed, but the directionality of the effects was different from our hypothesis. Specifically, increased activity in relation to longer hearing aid experience was found in the cerebellum, right precentral gyrus, right central operculum, right posterior cingulate cortex, and right middle occipital gyrus. Whereas some of these regions are related to the speech processing network, specifically the central operculum in the inferior frontal gyrus^10,58^, the majority of these regions are not typically associated with speech processing—although connections to speech processing regions by means of for example the fronto-occipital fasciculus exist^10^. The cerebellum has previously been associated with regulating language^59^, auditory processing^60,61^ and more specifically processing of auditory complex sentences^34,62^. Similarly, whereas typically associated with visual processing, occipital areas have previously been associated with auditory processing^12,28,34,63,64^—although most of these studies^12,34,63,64^ found peak activities located in the inferior rather than the middle occipital gyrus. Notably, the correlation of hearing aid experience with activity in the right middle occipital gyrus did not survive outlier correction, and should therefore be interpreted with caution. The right posterior cingulate cortex is part of the limbic system and has also previously been associated with language processing^14,65,66^.

Finally, the involvement of frontal regions was predicted based on previous work^13,14,44,45^ and by findings of Habicht et al.^28^. Precentral activity, as found in our study, is traditionally associated with motor activity, but has commonly been found to play a role in language processing as well^10^. However, Habicht et al.^28^ found decreased rather than increased activity in right frontal areas and in left and right cerebellar areas for hearing aided compared to unaided listeners. The same study found increased activity in several brain regions with longer hearing aid experience, but these regions—lingual gyri and the left precuneus—show little overlap with the regions identified in the current experiment. Thus, we could not replicate the findings of Habicht et al.^28^. However, similarly to the findings of Habicht et al.^28^ we found increased activity in several brain regions correlating with longer hearing aid experience. As increased activity is generally seen as a sign of processing effort, at first glance these findings seem surprising. One possible explanation could be that as hearing aid users were tested in the MRI scanner *without* their hearing aids, long-term users were affected by the absence of their hearing aid most, leading to more effortful processing of complex sentences. As this is just one possible explanation, it is clear that more research on (neural) speech processing in hearing aid users is needed. In light of this, the current study can be seen as providing a new list of potential brain regions to further investigate in relation to hearing aid use and experience.

Interestingly, the effect of hearing aid experience in our experiment was only observed in the most complex sentence condition. Similar but less salient patterns (i.e. not significant in whole-brain analyses) were observed for the other complex sentences, but not for the canonical sentences*.* This has important consequences for future research on the influence of hearing aid experience, which often does not include complex sentences (but see^28^). This finding is likely related to the more taxing nature of more complex sentences^18,31,32^, which are therefore more likely to invoke compensatory mechanisms. Overall, this research gives one of the first indications of changes in cortical recruitment associated with longer hearing aid experience, but explicitly calls for further research to confirm its findings. These neural adaptations illustrate that the brain adapts to the use of hearing aids and that this influences higher-level language processing (i.e. the processing of complex sentences).

Lastly, we expected improved comprehension of complex sentences following previous findings of improvements in speech identification following hearing aid fitting [e.g.,^23^]. No behavioral effects were found in the current study, although we do not interpret this as evidence of an absence of effects of hearing aid experience on sentence processing, especially as effects were found at a neural level. In our view, these results merely signal that evaluating processing at a neural level is a more sensitive measure than comprehension at a behavioral level; the latter might be indistinguishable in different individuals even though compensatory cortical neuroplasticity is involved. Neural measures, in contrast, can pick up changes in processing that are not necessarily reflected in performance.

## Conclusion

In the current study, we investigated the influence of hearing loss and hearing aid experience on sentence processing. To this end, we ran an fMRI study in which participants processed simple and complex auditory sentences. In comprehension, we found no influence of hearing loss severity or hearing aid experience on sentence comprehension. In neural processing, however, hearing loss severity was associated with increased activity in left superior frontal and left anterior insular areas, but only when processing complex object-before-subject sentences. Longer hearing aid experience was associated with the recruitment of several areas outside of the core speech processing network, including the cerebellum, right precentral gyrus, and right cingulate cortex when processing the most complex sentences used in the experiment. We interpret these findings as illustrating that the brain adapts to the use of hearing aids and that this influences language processing, indicating that it is important to investigate the adaptive capacity of the brain with regard to the effect of hearing aid use and language processing.

## Supplementary Information

Supplementary Information

## Data Availability

The datasets analyzed during the current study are available from the corresponding author on reasonable request.
