# Supplementary material for: Effects of age-related hearing loss and hearing aid experience on sentence processing
Source: Sci Rep. 2021 Mar 16;11:5994. doi: 10.1038/s41598-021-85349-5 (PMC7971046; doi:10.1038/s41598-021-85349-5)
Supplement: Supplementary file 1 — Supplementary Information [file 41598_2021_85349_MOESM1_ESM.pdf]

Effects of age-related hearing loss and hearing aid experience on sentence processing

Supplementary Information

Margreet Vogelzang, Christiane M. Thiel, Stephanie Rosemann, Jochem W. Rieger, & Esther Ruigendijk

Supplementary Table T1: Maxima of brain regions associated with the processing of canonical sentences.

Supplementary Figure F1: Neural activity associated with the processing of canonical sentences.

Supplementary Figure F2: Associations between neural activity and hearing loss.

Supplementary Figure F3: Associations between neural activity and years of hearing aid experience.

Supplementary Table T1: Maxima of brain regions (MNI coordinates) for the increased neural activity for the processing of sentences with canonical word order compared to the silent baseline (SVAO > baseline; L = Left; Inf. = infinite; Family-Wise Error corrected on the cluster level, threshold of $p < 0.05$).

Peak coord. (x,y,z)	Z-value	Cluster size	Brain region
**(-60, 10, -4)**	**Inf.**	**72955**	**L superior temporal gyrus**
(-8, 8, 54)	Inf.		L supplementary motor cortex
(-46, -2, 44)	Inf.		L precentral gyrus

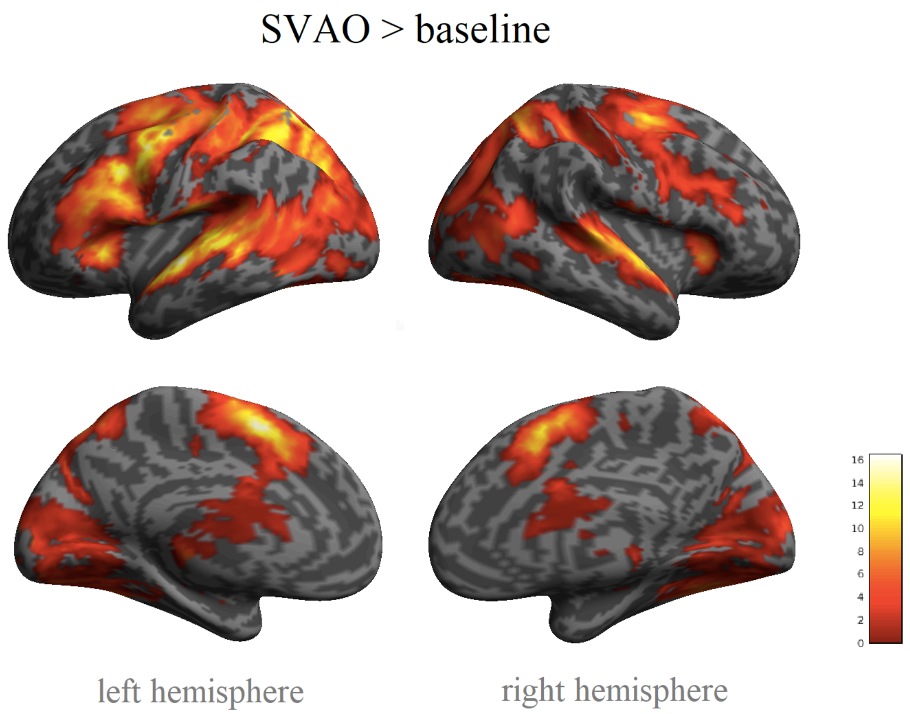

Supplementary Figure F1: Neural activity associated with the processing of canonical sentences.

Increased neural activity for the processing of sentences with canonical word order compared to the silent baseline (SVAO > baseline; Family-Wise Error corrected on the cluster level, threshold of $p < 0.05$). Inflated brain images were created with SPM12 (http://www.fil.ion.ucl.ac.uk/spm); the colors reflect the t-values from the T-contrasts.

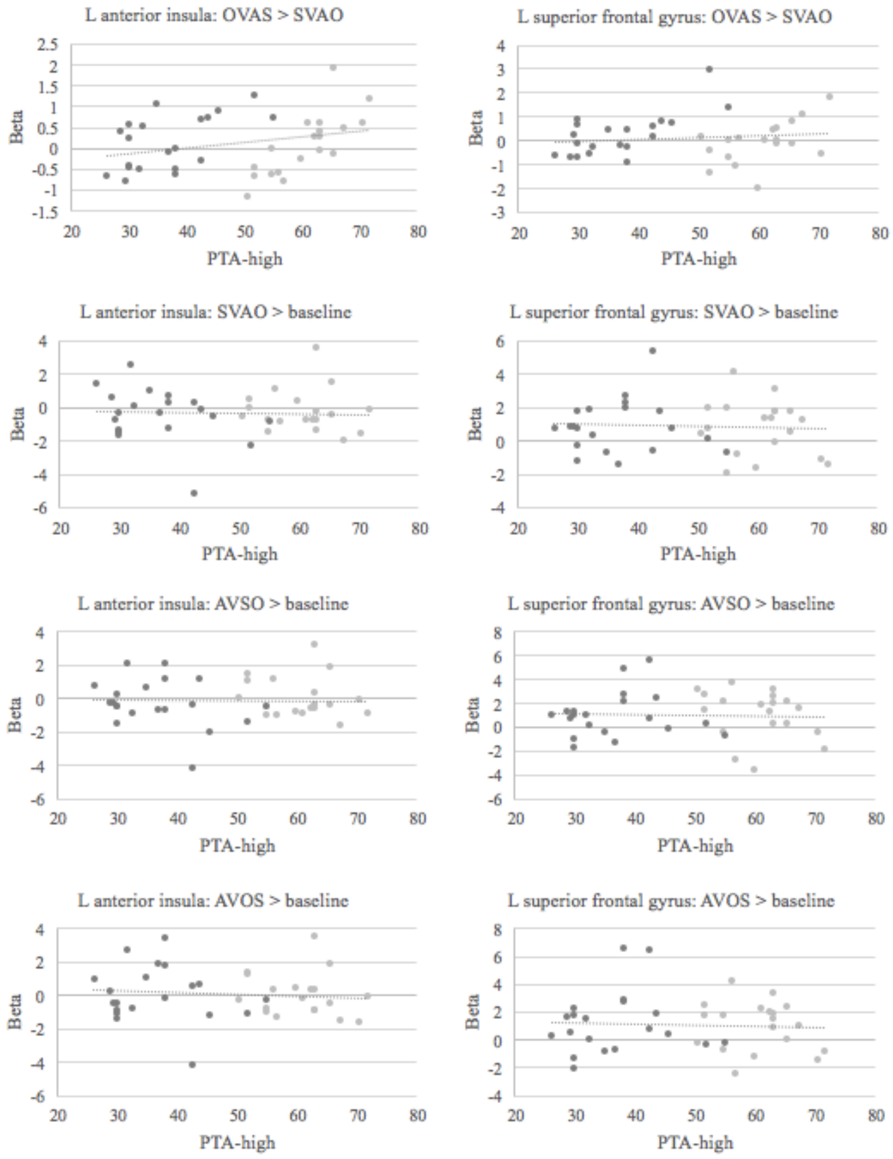

Supplementary Figure F2: Associations between neural activity and hearing loss.

Plots of the (exploratory) associations between neural activity and hearing loss (PTA-high) in the four sentence contrasts at the peak coordinates from Table 4 in the manuscript (hearing aid users in light grey, non-hearing aid users in dark grey). Associations for the contrast shown in the top row (OVAS > SVAO) reached significance in the whole-brain analyses.

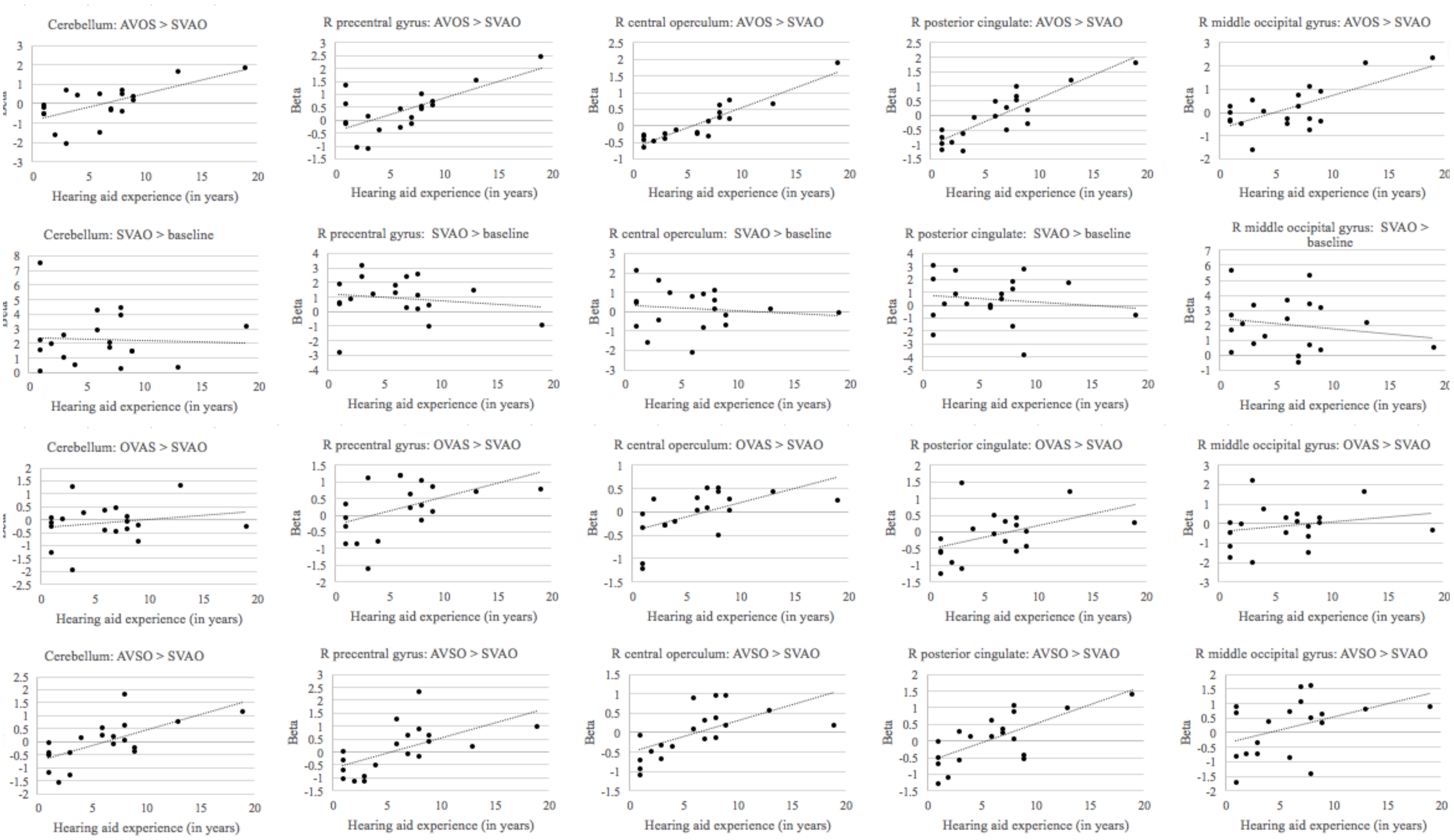

Supplementary Figure F3: Associations between neural activity and years of hearing aid experience.

Plots of the (exploratory) associations between neural activity and years of hearing aid experience in the four sentence contrasts at the peak coordinates from Table 5 in the manuscript. Associations for the contrast shown in the top row (AVOS > SVAO) reached significance in the whole-brain analyses.
